# Molecular study on the *carAB* operon reveals that *carB* gene is required for swimming and biofilm formation in *Xanthomonas citri* subsp. *citri*

**DOI:** 10.1186/s12866-015-0555-9

**Published:** 2015-10-23

**Authors:** Tao Zhuo, Wei Rou, Xue Song, Jing Guo, Xiaojing Fan, Gicharu Gibson Kamau, Huasong Zou

**Affiliations:** College of Plant Protection, Fujian Agriculture and Forestry University, Fuzhou, 350002 China; Hebei Institute of Engineering Technology, Shijiazhuang, 050091 China; School of Agriculture and Biology, Shanghai Jiao Tong University, Shanghai, 200240 China

**Keywords:** *Xanthomonas citri* subsp.*citri*, *carAB* operon, Swimming, Biofilm

## Abstract

**Background:**

The *carA* and *carB* genes code the small and large subunits of carbamoyl-phosphate synthase (CPS) that responsible for arginine and pyrimidine production. The purpose of this work was to study the gene organization and expression pattern of *carAB* operon, and the biological functions of *carA* and *carB* genes in *Xanthomonas citri* subsp. *citri*.

**Methods:**

RT-PCR method was employed to identify the full length of *carAB* operon transcript in *X. citri* subsp. *citri*. The promoter of *carAB* operon was predicted and analyzed its activity by fusing a GUS reporter gene. The swimming motility was tested on 0.25 % agar NY plates with 1 % glucose. Biofilm was measured by cell adhesion to polyvinyl chloride 96-well plate.

**Results:**

The results indicated that *carAB* operon was composed of five gene members *carA-orf-carB-greA-rpfE*. A single promoter was predicted from the nucleotide sequence upstream of *carAB* operon, and its sensitivity to glutamic acid, uracil and arginine was confirmed by fusing a GUS reporter gene. Deletion mutagenesis of *carB* gene resulted in reduced abilities in swimming on soft solid media and in forming biofilm on polystyrene microtiter plates.

**Conclusions:**

From these results, we concluded that *carAB* operon was involved in multiple biological processes in *X. citri* subsp. *citri*.

**Electronic supplementary material:**

The online version of this article (doi:10.1186/s12866-015-0555-9) contains supplementary material, which is available to authorized users.

## Background

Carbamoyl-phosphate synthase (CPS) catalyzes biocarbonate, ATP and glutamine to produce carbamoyl-phosphate, which serves as a precursor for synthesis of arginine and pyrimidine nucleotides in an alternative pathway [1]. In eukaryotes, CPS is responsible for removal of the excess and potentially neurotoxic ammonia via the urea cycle [2]. CPS deficiency results in an autosomal recessive disorder of urea cycle that leads to life threatening hyperammonemia [3]. Its primary localization in mitochondria makes it an ideal marker for mitochondrial damage and also during subacute phase of cecal ligation in the liver and in puncture sepsis [4, 5].

In most prokaryotes, CPS is composed of one minor and one major subunit. The smaller subunit CPSI is coded by the *carA* gene, and the larger subunit CPSII is coded by the *carB* gene [6–9]. CPSII mutant retains the ability to invade hosts but do not replicate in vivo [10]. CPSI and CPSII are proposed to combine with each other to form a tetrameric (αβ)_4_ protein, possessing an ammonia tunnel and a carbamate tunnel [11]. In prokaryotes, urea cycle does not occur and therefore the biological function of CPS is for arginine and the pyrimidine production.

The expression of carbamoyl phosphate synthetase is controlled by various metabolites along the pathways for pyrimidine and arginine synthesis. There exist two promoters in the promoter region of *carAB* operon in *Eschericha coli* [12, 13]. The upstream promoter P1 responds to pyrimidine and the downstream promoter P2 responds to arginine. The P1 is regulated by at least five transcription factors IHF, CarP, PyrH, PurR and RutR [14–16], while the P2 promoter is under the control of an arginine sensor ArgR [17]. Disruption of the repressors increases the *carAB* expression levels, which is positively correlated with pyridine production [18]. A recent work infers that *carAB* operon in *E. coli* is regulated through high intracellular levels of UTP that promote reiterative transcription to add extra U residues to the 3′ end of a nascent transcript during transcription initiation [19].

In plant bacterial pathogens, CPSI and CPSII are universal [8, 9, 20, 21]. However, their biological functions are not well understood. This research was carried out on *X. citri* subsp. *citri*, the causal agent of citrus canker disease. This is a severe bacterial disease affecting most commercial citrus cultivars grown in subtropical producing regions of the world. In the genome sequence of *X. citri* subsp. *citri* strain 306, XAC1861 and XAC1862 encode the small and large subunits CPSI and CPSII, respectively [9]. CPSII is thought to be involved in type II and type III secretion systems [9]. In this study, we reported the full-length transcript of *carAB* operon in *X. citri* subsp. *citri*. Our data showed that five genes *carA*, *orf*, *carB*, *gerA* and *rpfE* formed the *carAB* operon. The promoter was sensitive to the CPS-catalyzed intermediate and their final products namely glutamic acid, uracil and arginine. In addition, the loss of CPSII resulted in the phenotypic alterations in bacterial growth, swimming motility and biofilm formation. These supported the idea that *carAB* operon was involved in multiple biological processes in *Xanthomonas citri* subsp. *citri.*

## Results

### The *carAB* operon was composed of *carA*, *orf*, *carB*, *gerA* and *rpfE* genes

**Fig. 1:**
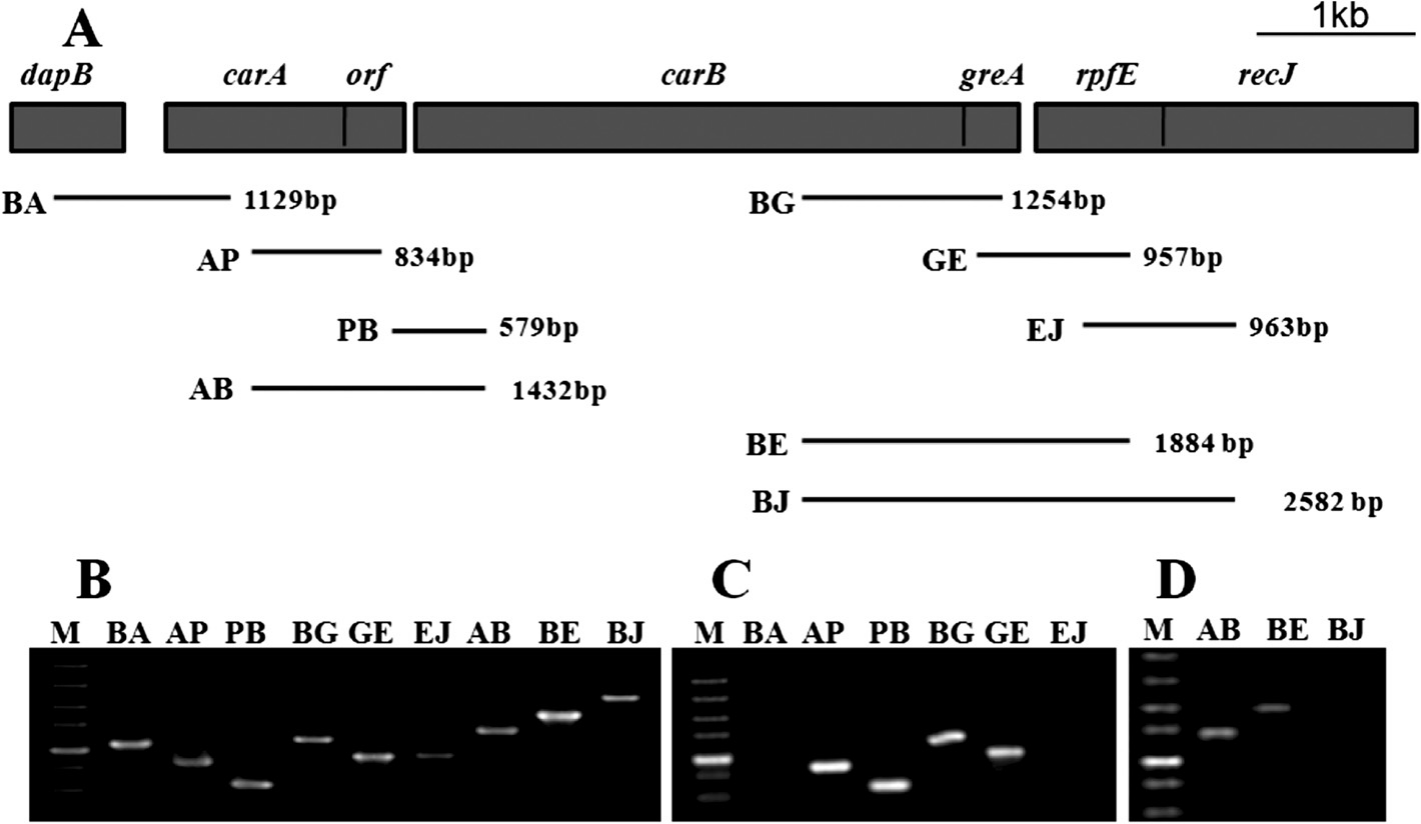
Schematic representation of the *carAB* operon and the RT-PCR strategy. **a** The grey rectangles depicted the open reading frames of the operon and their lengths in base pairs. The arrows represent the sizes and approximate locations in the PCR analysis with primer sets. All forward and reverse primers were gene-specific. **b** PCR products by using gDNA as template. **c** and **d** PCR products from first strand cDNA. The DNA marker was DL5000

In the genome of *X. citri* subsp. *citri* strain 306*,* the CPS small subunit *carA* gene is annotated as ORF XAC1861, which is downstream of the dihydrodipicolinate reductase *dapB* gene (XAC1860). Even though the *carB* gene is annotated as XAC1862, there exists a 444-bp putative ORF between *carA* and *carB* genes. The nucleotide sequence of this *orf* overlaps one base at the 3′ end of *carA* gene. The *greA*, *rpfE* and *recJ* are consequently localized downstream of *carB* gene (Fig. 1a). Twelve specific primers were first applied to produce 1129 bp BA, 834 AP, 579 bp PB, 1254 bp BG, 957 GE and 963 bp EJ fragments by using gDNA as template (Fig. 1b; Additional file 1: Table S1). To detect the full length of the mRNA of *carAB* operon, the corresponding primer sets were applied to run RT-PCR from first strand of cDNA. Figure 1c showed that the primer combination D.F/A.R could not amplify the 1129 bp BA fragment covering partial *dapB* and *carA* genes. Meanwhile, the primer combination E.F/J.R did not produce the 963 bp EJ fragment containing partial *rpfE* and partial *recJ* genes. By contrast, the left AP, PB, BG and GE fragments were successfully amplified from the reverse transcript cDNA. For further confirmation, primer combinations A.F/B.R, B.F/E.R, B.F/J.R were used to amplify the desired 1423 bp AB, 1884 bp BE and 2582 bp BJ fragments from cDNA. We successfully amplified the AB and BE fragments, but not the 2582 bp BJ fragment (Fig. 1d). This indicated that *carA*, *orf*, *carB*, *gerA* and *rpfE* were localized within *carAB* operon.

### The transcription of *carAB* operon was suppressed by glutamic acid, uracil and arginine

**Fig. 2:**
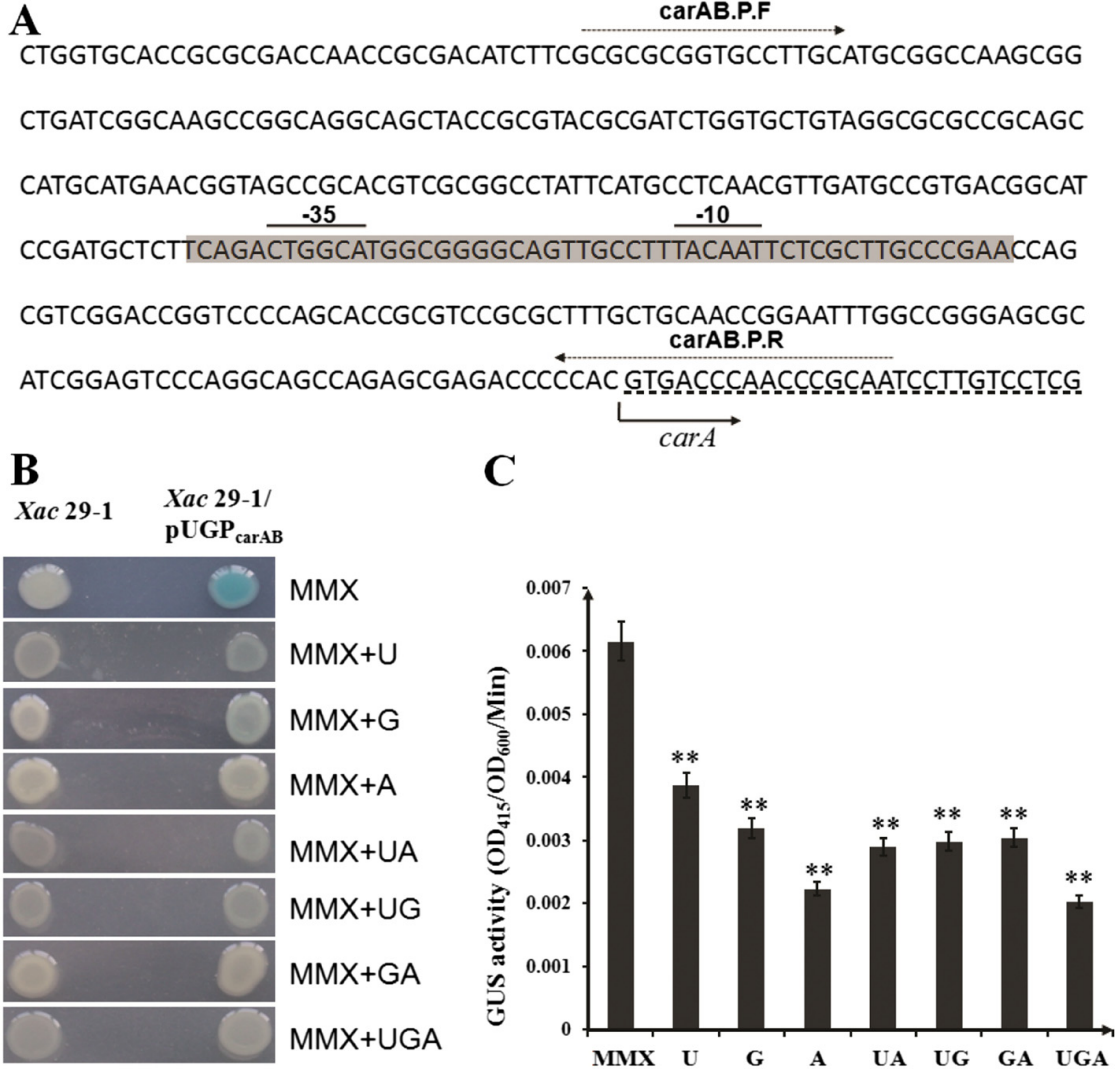
Analysis of *carAB* operon promoter and its activity. **a** Nucleotide sequence of *carAB* operon promoter region. The promoter sequence was shaded in grey colour, and the −35 and −10 promoter elements were overlined. Dotted-line arrows indicated the forward and reverse primers for promoter cloning. The transcription initiation was shown by a black arrow. **b** The promoter-monitored GUS activities on MMX plates. The applied substrates uracil, glutamic acid and arginine were indicated by the first letter as U, G and A. U, uracil; G, glutamic acid; A, arginine; UA, uracil and arginine; UG, uracil and glutamic acid; GA, glutamic acid and arginine; UGA, uracil, glutamic acid and arginine. **c** Quantification of GUS activities in MMX liquid media. The experiment was repeated three times, and similar results were obtained. The asterisks in horizontal data column indicate significant differences at *P* = 0.01 by *t* test

We analyzed 0.5 kb DNA sequence upstream of *carA* gene in Neutral Network Promoter Prediction (http://fruitfly.org/seq_tools/promoter.html), and predicted one putative promoter at 102 bp from the translation start codon of *carA* gene (Fig. 2a). The promoter region was fused with GUS reporter gene and cloned into cosmid pUFR034 to monitor promoter activity. The recombinant construct pUGP_carAB_ was introduced into wild type *X. citri* subsp. *citri* strain *Xac* 29–1. Since glutamic acid, uracil and arginine are the biosynthetic products of CPS, 0.5 % of each one of them was added into minimal medium plates as substrate for promoter activity examination. On solid MMX plates, the GUS activities were clearly viewed from minimal media at 3 days after inoculation. All the three products had suppression effect on GUS activities (Fig. 2b). Further quantification experiments revealed that GUS activity was suppressed in the presence of uracil and glutamic acid by 37 and 48 % respectively. In the media with arginine, it was just 36 % of that in minimal media (Fig. 2c). These data suggested that the expression of *carAB* operon was suppressed by glutamic acid, uracil and arginine.

### *carB* gene was required for bacterial biofilm formation

**Fig. 3:**
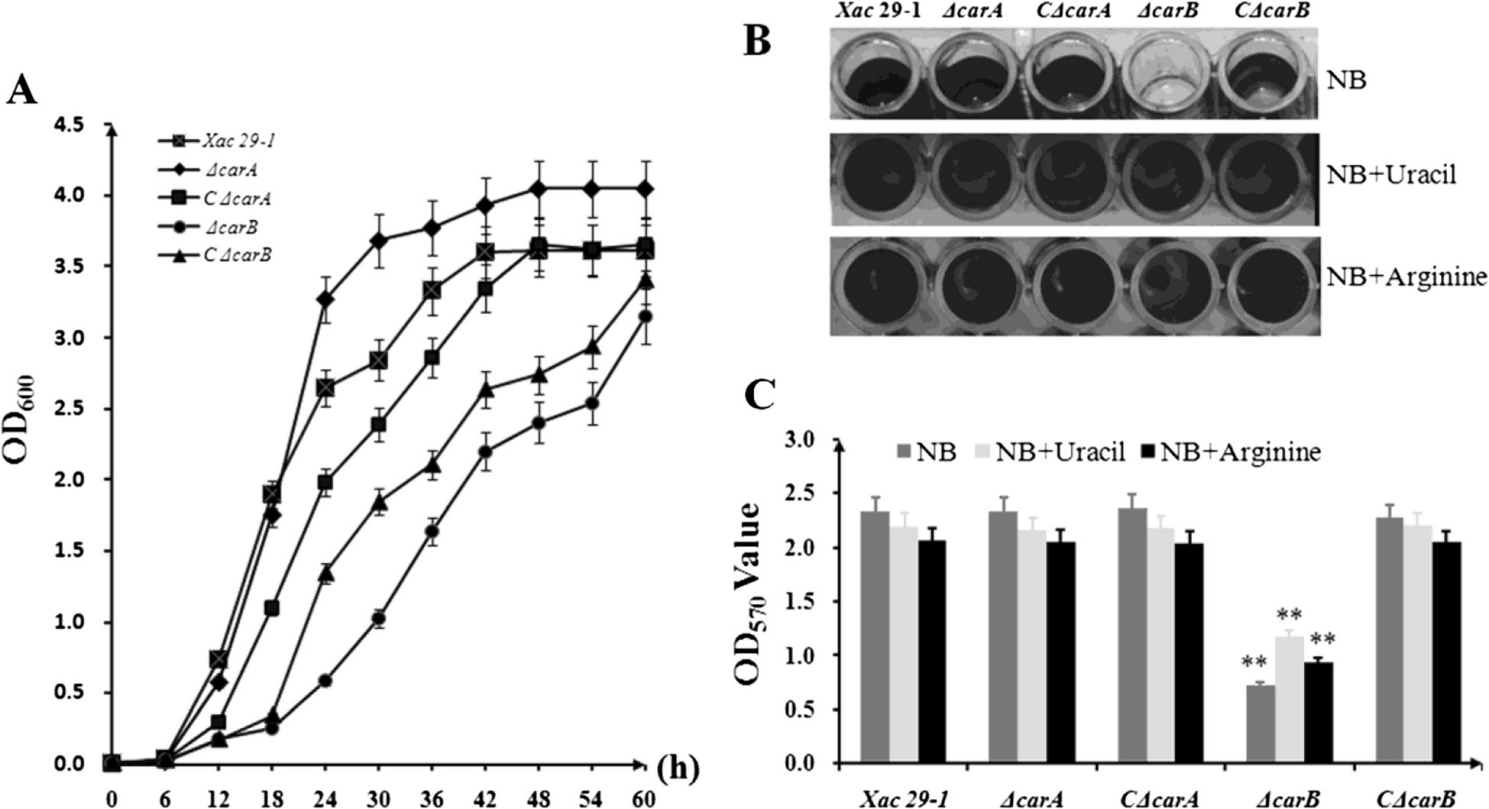
Bacterial growth and biofilm formation. **a** Growth curve of *ΔcarA* and *ΔcarB* mutants in NB media. **b** Biofilms of *ΔcarA* and *ΔcarB* mutants formed in a microtiter plate and stained with crystal violet. **c** Quantitative measurements of biofilm formation. The experiments were repeated three times. The asterisks in horizontal data column indicate significant differences at *P* = 0.01 by *t* test

In a previous study, we constructed *carA* and *carB* gene mutants and revealed that only *carB* played a role in bacterial pathogenicity [9]. To detect their roles in biofilm formation, deletion mutants *ΔcarA* and *ΔcarB* were cultured in NB media to examine whether the mutations had any effect on bacterial growth (Fig. 3a). Results showed that loss of CPSI slightly increased bacterial growth a phenotype which was also observed in bacteria growing in MMX medium [9]. In contrast, the loss of CPSII led to remarkably reduced growth speed. At every observation time point after inoculation, the cell density of *ΔcarB* was lower than that of the wild type and *ΔcarA* though its OD_600nm_ value reached 2.0 at 42 h after inoculation and increased to 3.0 at 60 h. For biofilm formation tests, we cultured all the strains to stationary growth stage, and adjusted the OD_600nm_ to 1.0. The *ΔcarA* mutants showed no distinct difference from wild type strain in biofim formation and the cells were tightly adhered to polystyrene microtiter plate (Fig. 3b). By contrast, the *ΔcarB* showed a 70 % decrease in biofilm formation. However, when the strain was complemented, its ability to form biofilm was restored to the level of the wild type strain (Fig. 3c). The addition of exogenous uracil and arginine partially restored biofim formation in *ΔcarB* mutant (Fig. 3b-c).

### *carB* gene was involved in cell swimming

**Fig. 4:**
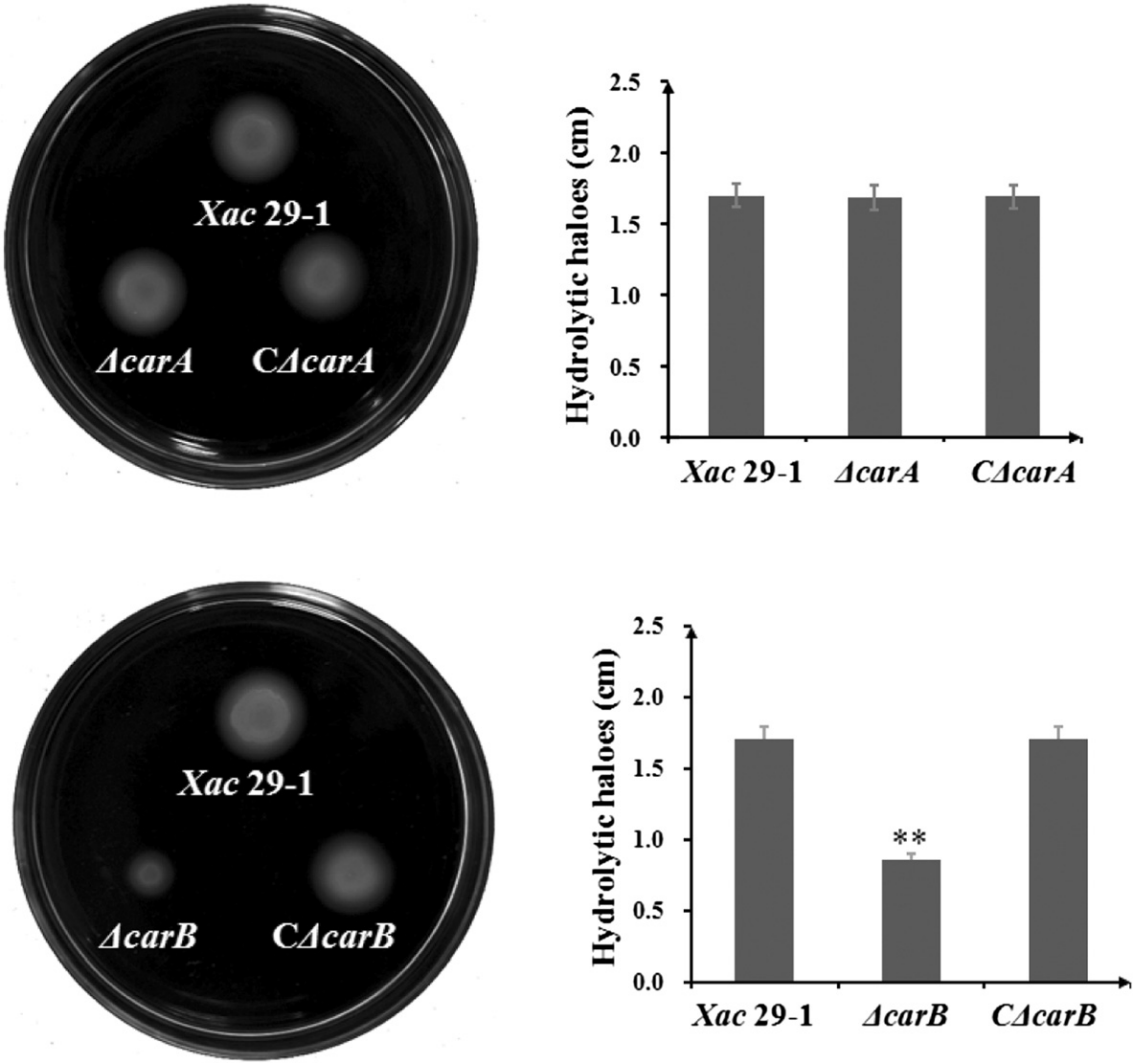
Swimming patterns of *ΔcarA* and *ΔcarB* mutants in NYG medium. The swimming motility was measured from the diameter of each colony at 2 days post inoculation. The tests were repeated three times. The asterisks in horizontal data column indicate significant differences at *P* = 0.01 by *t* test

The cell motility ability was studied on 0.25 % agar NYG plates supplemented with 1 % glucose. Two days after incubation at 28 °C, the diffusion area of the wild type strain and *ΔcarA* mutant were clearly viewed near the colonies and there was no difference in their swimming ability. By contrast, mutant *ΔcarB* showed a considerable reduction in its motility (Fig. 4). The diameters derived from *ΔcarB* mutant reduced by over 50 % when compared with wild type. The complemented mutant strains showed a restoration of their motility confirming that the loss of CPSII affected flagellar-dependent swimming (Fig. 4).

## Discussion

In our previous study, the mutations in *carA* and *carB* gene resulted in diverse effects on bacterial pathogenicity [9]. The *ΔcarA* retained the ability to produce citrus canker on host plant and to induce hypersensitive response on nonhost plants, while the *ΔcarB* mutant abolished neither pathogenicity nor extracellular enzyme activity [9]. In this work, we confirmed that the mutation in *carA* gene resulted in slightly increased bacterial growth speed. The maximum OD_600nm_ value at stationary stage in NB broth was higher than wild type and was consistent with the results of our previous work that investigated effect of culturing bacteria in minimal media MMX [9]. Loss of CPSII led to a remarkably reduction of nitrogen and carbon resource assimilation indicating *carB* gene was more important than *carA* in nucleotide and amino acid metabolism in *X. citri* subsp. *citri.* Additionally, the loss of CPSII led to a reduction of cell swimming ability by 50 % and biofilm formation was reduced by 70 %. This suggested that *carB* gene played the role in canker development during the early infection stage.

In the model prokaryote *Eschericha coli*, dihydrodipicolinate reductase *caiF* is downstream of *carB* and has the same transcription orientation as *carA* and *carB* genes (Additional file 2: Figure S1). It is transcribed as a monocistronic mRNA under anaerobiosis independent of the presence of carnitine [22]. Solid evidence has been presented for the organization of *carAB* operon in *Pseudomonas aeruginosa* which was found to contain four gene members namely *carA*-*orf*-*carB*–*greA* [23]. The genetic function of *greA* gene has been well demonstrated [23]. In *X. citri* subsp. *citri*, the genetic organization of *carAB* operon is similar to that in *P. aeruginosa* (Additional file 2: Figure S1). However, it also has a putative protein coding ORF between *carA* and *carB*, even though both *orfs* showed no similarity (data not shown). In addition to the *greA* which is located downstream of *carB* gene, there are two genes *rpfE* and *recJ* having the same transcription orientation with *carA* and *carB* genes [20]. Through RT-PCR method, we demonstrated that *X. citri* subsp. *citri* has an extra fifth member in its *carAB* operon namely the *rpfE* gene. Thus, the *carAB* operon in *X. citri* subsp. *citri* is different from those found in *P. aeruginosa* and *E. coli.*

The transcriptional elongation factor GreA induces the endonucleolytic cleavage that occurs at the 3′ ends of arrested quarternary transcription complexes [23]. Mutation in *greA* gene results in loss of growth ability in minimal medium with arginine and nucleosides, but the mutant grows well in rich media [23]. The *rpfE* is required for full virulence, as well as swarming motility and production of cellulase and extracellular polysaccharide. In culture, *rpfE* mutant strain is unable to efficiently utilize sucrose or xylose as carbon sources [24]. Pyrimindine, the final product catalyzed by CPS, is involved in biofilm formation [25, 26]. Exogenous pyrimindine restores cellulose production in *carB* mutant, which is one of the extracellular adhesion and cell aggregation factors responsible for bacterial biofilm formation and maintenance [26]. Thus, the gene members in *carAB* operon are required for multiple cell life activities, including amino acid and nucleotide biosynthesis, cell motility, cellulose and extracellular polysaccharide production. Some of the processes are co-affected by the members in *carAB* operon, because CPSII and RpfE are both involved in cell motility and cellulose activity [9, 23].

There are two adjacent promoters for *carAB* operon in *E. coli* namely P1 and P2. The P1 promoter is located far from translation initiation site and is mainly regulated by pyrimidines while the P2 promoter which overlaps a pair of ARG boxes is regulated by arginine and the arginine repressor ArgR [13, 17]. A single promoter has been identified from *carAB* operon in *P. aeruginosa*, which is controlled by pyrimidines and arginine [7]. In this work, only one promoter was identified from *carAB* operon in *X. citri* subsp. *citri*. The promoter activity was suppressed by pyrimidines, arginine and glutamic acid, which was similar with the result obtained from *P. aeruginosa.* It appeared that the regulation mechanism of *carAB* operon in *X. citri* subsp. *citri* resembled that in *P. aeruginosa,* but differed from that in *E. coli*.

## Conclusions

In this study we found out that *X. citri* subsp. *citri carAB* operon was made up of five genes *carA, orf, carB, gerA* and *rpfE*, and that its transcription was suppressed by glutamic acid, uracil and arginine. We also found out that the loss of the large subunit CPSII resulted to phenotypic alterations in bacterial growth, biofilm formation and swimming.

## Methods

### Bacterial strains, plasmids and culture conditions

Bacterial strains and plasmids used in this study are listed in Table 1. The *X. citri* spp. *citri* strains 29–1 (*Xac* 29–1) were cultivated in NB nutrient broth or NB with 1.5 % agar at 28 °C [9]. *E. coli* strains were cultured in Luria-Bertani medium (LB) at 37 °C. Three antibiotics were used at the following concentrations: ampicillin (Ap), 100 μg/ml; kanamycin (Km), 25 μg/ml and Gentamycin (Gm), 50 μg/ml.

**Table 1:** Strains and plasmids used in this study

Strain or plasmid	Relevant characteristics	Source
strains		
*Escherichia coli*		
DH5 α	*Φ901acZΔm15*, *recA1*	Invitrogen
*Xanthomonas citri* subsp. *citri*		
*Xac* 29-1	Wild-type	This lab
*Xac* 29-1/pUGP_carAB_	*Xac* 29*–*1 carrying pUGP_carAB_	This work
*ΔcarA*	A *carA* knock-out mutant of strain *Xac* 29-1	[9]
*ΔcarB*	A *carB* knock-out mutant of strain *Xac* 29-1	[9]
C*ΔcarA*	Gm^r^, *ΔcarB* harboring pBBR1MCS-5 expressing *carA* gene under *wxacO* promoter	[9]
C*ΔcarB*	Gm^r^, *ΔcarB* harboring pBBR1MCS-5 expressing *carB* gene under *wxacO* promoter	[9]
Plasmids		
pUFR034	Km^r^, *IncW*, *Mob(p), Mob* ^*+*^ *, LacZa* ^*+*^, PK2 replicon, cosmid	[29]
pUGP_carAB_	Km^r^, *carAB* operon promoter and *gusA* gene fused in pUFR034 vector	This work

### RT-PCR

To detect the full length mRNA of *carAB* operon, specific primers (Additional file 1: Table S1) were designed according to genome sequence information. After wild type *Xac* 29–1 was cultured in liquid NB broth, RNAs were extracted from cells by the RNA prep pure Cell/Bacteria Kit (Tiangen Biotech, Beijing, China). The total RNAs were quantified by measuring the OD_260_/OD_280_ ratio and then analyzed by gel electrophoresis to find out whether they were intact. To ensure genomic DNA was not contaminated, the PrimeScript™ RT reagent Kit with gDNA Eraser (TaKaRa-bio, Dalian, China) was used before reverse transcription. 2 μg total RNA was reverse transcribed to first strand cDNA by AMV reverse transcriptase (TaKaRa-bio, Dalian, China). The PCR thermal cycle consisted of an initial denaturation at 94 °C for 5 min, 32 cycles of DNA denaturation at 94 °C for 30 s, primer annealing at 52 °C for 40 s, and primer extension at 72 °C for 1 min, and followed by a final elongation step at 72 °C for 10 min. The expression of *gyrA* was used as a control to verify the quality of cDNA.

### GUS activity assays

To construct the promoter β-glucuronidase (GUS) fusion, a 336 bp promoter region upstream of *carA* gene was PCR amplified from genome DNA by primer carAB.P.F and carAB.P.R (Additional file 1: Table S1). The *gusA* gene was amplified by primer GUSA.F and GUSA.R and ligased into pUFR034, together with the 336 bp promoter fragment (Additional file 1: Table S1). Recombinant pUGP_carAB_ was introduced into wild type *Xac* 29–1 to generate *Xac* 29-1/pUGP_carAB_. The strain was cultured in NB until OD_600nm_ reached 0.8. After centrifugation at 6000 rpm for 10 min at 4 °C, the cell pellets were re-suspended in NB broth to OD_600nm_ = 1.0. About 1.5 μl of the cell suspension was added to MMX medium plates containing 50 μg/ml of X-gluc. To assess the effects on *carAB* transcription, 0.5 % of glutamic acid, uracil or arginine was applied to MMX medium plates. At 3 days after inoculation, colony color on plates was observed to determine GUS activities. In the parallel experiments, 3 ml liquid MMX media was used to culture the cells at 28 °C for 12 h induction. The cells were then collected for GUS activity analysis [27]. GUS activities were determined at 30 min intervals for 3 h by measuring absorbance at 415 nm (*A*_415_) using *p*-nitrophenyl-D-glucuronide as the substrate. One unit was defined as 1 nmol of 4-methyl-umbelliferone produced per min per bacterium. Assays were repeated three times independently.

### Swimming motility assay

The swimming motility was performed as described previously [28]. In brief, the cultured *X. citri* subsp. *citri* strains were suspended in sterile distilled water to a final concentration of OD_600nm_ = 1.0. 1.5 μL of each cell sample was dropped to 0.25 % agar NY plates with 1 % glucose. The plates were stationary incubated at 28 °C. The motile ability was measured from the diameter of each colony 2 days post cultivation. The tests were repeated three times.

### Determination of bacterial growth

The cultured cells were washed twice with sterilized water, and then re-suspended in sterilized water to OD_600nm_ = 1.0. The re-suspended cells were subcultured (1:100) in NB broth media. The OD_600nm_ values were tested after every 6 h post sub-culturing. All the experiments were repeated at least three times.

### Biofilm assays

Biofilm was measured by cell adhesion to poly (vinyl chloride) 96-well plate (Falcon 353913, Becton Dickinson). All the cultured strains were re-suspended in NB to an OD_600nm_ of 1.0. To assess the effects of uracil and arginine on biofilm formation, 0.5 % of each chemical was applied to NB medium. Typically, 100 μl cell suspensions were dropped into one well of a microtiter plate. The plates were sealed with plastic wrap and incubated without shaking for 72 h at 28 °C. Bacterial adhesion was measured after repetitive washing of the plates and staining with 1 % crystal violet for 15 min at room temperature. Excess stain was removed by gently washing with distilled water, and the crystal violet stain was solubilized by the addition of 150 μl of 95 % ethanol to each well. Crystal violet was then quantified with a microplate reader at *A570* absorption wavelength. All the experiments were repeated at least four times and the average for each strain was checked by *T*-test.

## Additional files

Additional file 1: Table S1. Primers used in this study. (DOC 38 kb) Additional file 2: Figure S1. Alignment of *carAB* operons from *Xanthomonas citri* subsp. *citri*, *Pseudomonas aeruginosa* and *Escherichia coli*. The genetic information from each strain was based their genome information in GenBank (NC_003919.1, NC_002516.2 and NC_002655.2). (DOC 41 kb)

## Abbreviations

CPS: Carbamoyl-phosphate synthase
CPSI: Carbamoyl-phosphate synthase small subunit
CPSII: Carbamoyl-phosphate synthase large subunit
GUS: β-Glucuronidase
ORF: Open reading frame
RT-PCR: Reverse transcription polymerase chain reaction
