# Supplementary material for: Molecular study on the carAB operon reveals that carB gene is required for swimming and biofilm formation in Xanthomonas citri subsp. citri
Source: BMC Microbiol. 2015 Oct 23;15:225. doi: 10.1186/s12866-015-0555-9 (PMC4619228; doi:10.1186/s12866-015-0555-9)
Supplement: Additional file 1: Table S1. — Primers used in this study. (DOC 38 kb) [file 12866_2015_555_MOESM1_ESM.doc]

**Additional file 1: Table S1** Primers used in this study

| Primer | Sequence (5’-3’) | Description |
| --- | --- | --- |
| D.F | TGCGCTGGTCTGGGCGTCCAACTTC | A 1129bp fragment containing partial *dapB* and partial *carA* |
| A.R | TCCAGTTGTACGTGCTCTCGGTCGA |
| A.F | GACAGCTTTTTGGGTCTGGGTGCGT | A 834bp fragment containing partial *carA* and partial orf |
| P.R | CGACGATGGCGCTCGCAAGGCTGTG |
| P.F | TTGCGTGGATTGGGTGACTGCCGTG | A 579bp fragment containing partial *orf* and partial *carB* |
| B.R | GACGTTGTACTTCTCCAGCACCCCG |
| B.F | GGCTTGAACGTAGTCGGCCTGATGA | A 1254bp fragment containing partial *carB* and partial *greA* |
| G.R | CCAGGTAGGCGACGCTGACGACTTC |
| G.F | CGGCGCGCGAGCAACAGAGCTTTAT | A 957bp fragment containing partial *greA* and partial *rpfE* |
| E.R | GCCACCCCAGAACCACAGCGAATTG |
| E.F | GGCGATGCTGGTTTTCTGTTGGACG | A 963bp fragment containing partial *rpfE* and partial *recJ* |
| J.R | CCGGCTGCAACCCCGCCAGTTCGTC |
| gyrA.F | CTGGACCAACACTGACACTGAG | a 423bp *gyrA* gene |
| gyrA.R | GTCTCCTTAGAGTTCCCACCAT |
| GusA.F | ATAAAGCTTAGTCCCTTATGTTACGTCC | A 1831 bp *gusA* gene |
| GusA.F | TAAGAATTCTCATTGTTTGCCTCCCTGCTG |  |
| carAB.P.F | TTGAATTCGCGCGCGGTGCCTTGC | A 336 bp *carAB* promoter region |
| carAB.P.R | TTAAGCTTGCGGGTTGGGTCACGTGG |  |
|  |  |  |
