# Supplementary figures and images for: Molecular study on the carAB operon reveals that carB gene is required for swimming and biofilm formation in Xanthomonas citri subsp. citri
Source: BMC Microbiol. 2015 Oct 23;15:225. doi: 10.1186/s12866-015-0555-9 (PMC4619228; doi:10.1186/s12866-015-0555-9)

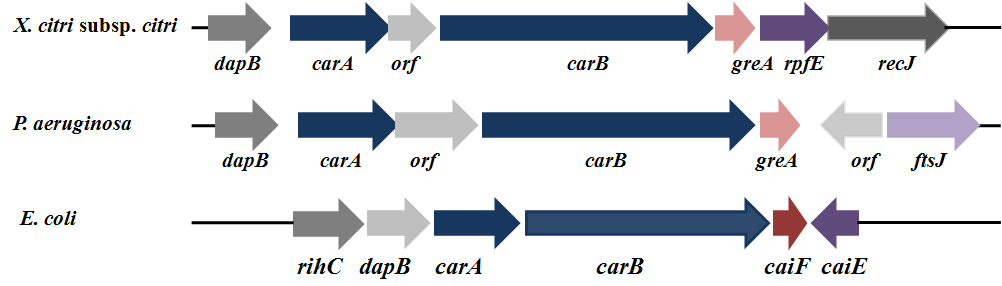
 **Additional file 2: Figure S1**

Supplement: Additional file 2: Figure S1. — Alignment of carAB operons from Xanthomonas citri subsp. citri, Pseudomonas aeruginosa and Escherichia coli. The genetic information from each strain was based their genome information in GenBank (NC_003919.1, NC_002516.2 and NC_002655.2). (DOC 41 kb) [file 12866_2015_555_MOESM2_ESM.doc]
